# Porous silicon functionalization for possible arsenic adsorption

**DOI:** 10.1186/1556-276X-9-508

**Published:** 2014-09-17

**Authors:** Ruth Fabiola Balderas-Valadez, Vivechana Agarwal

**Affiliations:** 1 CIICAp, UAEM, Av. Universidad 1001 Col. Chamilpa, Cuernavaca, Morelos CP 62210, Mexico

**Keywords:** Arsenic, Porous silicon, DMSA, Adsorption

## Abstract

Thiol-functionalized porous silicon (PS) monolayer was evaluated for its possible application in As (III) adsorption. Dimercaptosuccinic acid (DMSA) attached to mesoporous silicon via amide bond linkages was used as a chelate for As (III). Two different aminosilanes namely 3-(aminopropyl) triethoxysilane (APTES) and 3-aminopropyl (diethoxy)-methylsilane (APDEMS) were tested as linkers to evaluate the relative response for DMSA attachment. The aminosilane-modified PS samples were attached to DMSA by wet impregnation followed by the adsorption of As (III). Fourier transform infrared (FTIR) and X-ray photoelectron spectroscopy (XPS) have been used to identify the functional groups and to estimate the As (III) content, respectively. FTIR spectroscopy confirmed the covalent bonding of DMSA with amide and R-COOH groups on the nanostructured porous surface. XPS confirms the preferred arsenic adsorption on the surface of PS/DMSA samples as compared to the aminosilane-modified and bare PS substrates.

## Background

The development of improved heavy metal absorbent materials that enhance the metal specificity has been a continued objective for environmental remediation purposes [1,2]. Porous materials, with large surface area and specificity, can be used as metal concentrator for detection purposes in possible metal pollution sites to make the transport and handling of the samples easier and relatively safe. On the other hand, thiol (-SH) compounds have been extensively studied as chelators for ‘soft’ elements like Cd, Hg, and Pb due to their preferential formation of covalent bond with sulfur [3]. In this way, thiol-functionalized mesoporous silica had been tested as possible heavy metal absorbent for its great potential in environmental and industrial processes because of its large surface area and well-defined pore size and pore shape [2,4,5].

Specifically, it had been widely studied in medicine since World War II where 2,3-dimercaptopropan-1-ol (also known as British anti-Lewisite, BAL) was used to reverse the effects of the chemical weapon, lewisite (2-chloroethenyldichloroarsine) [6]. Since then, different chelates had been tested, e.g. 2,3-dimercaptosuccinic acid (DMSA); it has proved to be more efficient in comparison to the classical treatment for human metal intoxications provided for BAL [7]. DMSA molecule has been widely studied not only in the medicine field but also in the surface functionalization field due to its capacity to form strong complex as a metal chelating agent. The unbound -SH group of the molecule can be used to graft a biomolecule [8]. DMSA has been anchored to different materials with biological [9] protector agent [10] and metal adsorption purposes [11]. Particularly, in the field of adsorption, DMSA was attached to silica via amide bond to be tested on Hg, Cd, and Pb absorption with purpose of IR detection [3]. The procedure for DMSA anchorage uses an aminosilane molecule as linker, where the amine group reacts with the carboxylic group of DMSA and forms an amide bond.

Porous silicon (PS) has provided a platform for developing multipurpose devices such as chemical [12] and biological sensors [13] and enzymatic catalysis [14]. PS is an inorganic material produced with galvanostatic, chemical, or photochemical etching of crystalline silicon in the presence of hydrofluoric acid (HF). It is relatively easier to modulate the PS surface in order to enable the attachment of appropriate chemical agents needed for the assigned task (sensing, separation, catalysis). Besides, the chemical modification of the PS surface can be monitored by the extrinsic optical properties of the layer which can be used as a label-free optical interferometric-based sensor where changes in the optical reflectivity spectrum (refractive index change) due to non-specific binding events on the structure surface or compositional fluctuations in the sample matrix indicate the capture of a chemical or biological species [15-17]. PS interferometric-based detection consists, in general, of the pore wall-analyte immobilization which generates a change in the refractive index of the layer and is detected as a corresponding shift in the interference patterns (therefore the optical thickness (OT)) [18]. This method is referred as reflective interferometric Fourier transform spectroscopy (RIFTS) [19].

In this study, we investigated the adsorption on As (III) by a thiol-functionalized PS monolayer chip using DMSA as dithiol source. This chip design allows safer transport and handling of the sample with arsenic. The PS thiol functionalization was conducted testing two different aminosilanes, 3-(aminopropyl) triethoxysilane (APTES) and 3-aminopropyl (diethoxy)-methylsilane (APDEMS), as linkers to evaluate the relative response for DMSA attachment. Optical interferometric-based non-destructive PS detection was used for the characterization of PS-aminosilane-DMSA functionalization procedure. Fourier transform infrared (FTIR) spectroscopy and X-ray photoelectron spectroscopy (XPS) were employed to confirm the interactions of thiol-functionalized PS and As (III).

## Methods

3-(Aminopropyl) triethoxysilane (APTES, 98%), 3-aminopropyl (diethoxy)-methylsilane (APDEMS, 97%), dimercaptosuccinic acid (DMSA), *N*-(3-dimethylaminopropyl)-N′-ethylcarbodiimide hydrochloride (EDC) and 1 g/l arsenic(III) standard solution (NaAsO_2_) were purchased from Sigma-Aldrich and used as received.

**Figure 1:**
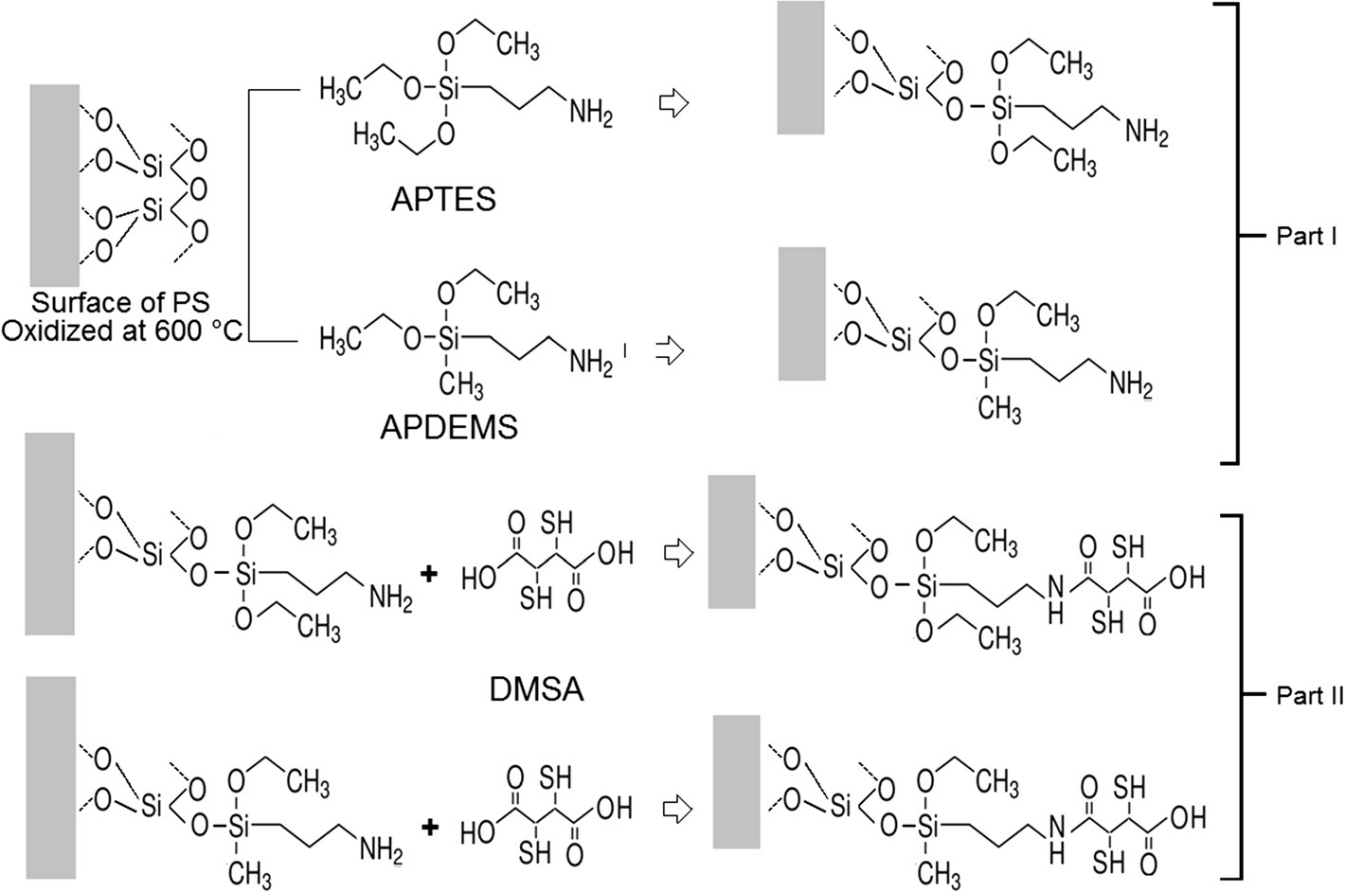
The process of thiol functionalization of oxidized PS via APTES and APDEMS.

PS monolayers were prepared by wet electrochemical etching of a silicon wafer using an electrolyte solution composed of HF (48%), ethanol (99.9%), and glycerol (98%) in a volumetric ratio of 3:7:1. The anodization process was realized in galvanostatic regime with a fixed current density of 50 mA/cm^2^ for 75 s at room temperature. A p++ type Si wafer with (100) crystal orientation and resistivity *ρ* = 0.002 to 0.005 Ω cm were used. Prior to aminosilane functionalization, PS was thermally oxidized in a furnace at 600°C, followed by its immersion in APTES or APDEMS solution at 5% in toluene at for a time period of 90 min. Then, the samples were rinsed three times each with toluene and ethanol/water in 1:1 (v/v) proportion and were allowed to dry in a stream of nitrogen gas. The silanized samples thus obtained were baked at 110°C for 15 min in order to enhance the aminosilane horizontal polymerization [20]. The aminosilane-modified PS samples were attached to DMSA by overnight wet impregnation in DMSA (5 mM) and EDC (5 mM) aqueous solution at room temperature. After this procedure, the samples were treated with ethanol/water in 1:1 (v/v) proportion, dried in a stream of nitrogen gas, and baked for 15 min at 110°C in order to dry completely. Finally, thiol-functionalized, aminosilane-functionalized, and oxidized PS samples were inundated with 1gr/l NaAsO_2_ solution at pH 2; these last two were taken as control samples. After 60 min of immersion, the samples were rinsed three times with dilute HCl at pH 2 and dried under the stream of nitrogen gas. Figure 1 shows the chemistry of the functionalization process involving the covalent attachment of DMSA through either APTES or APDEMS onto the oxidized PS surface.

The bonding on the PS pore walls was monitored by recording the changes in the reflectance spectrum of the dried porous layer during each modification step using a UV-Vis spectrophotometer (PerkinElmer Lambda 950, PerkinElmer, Waltham, MA, USA) coupled with universal reflectance accessory, with 5 × 5 mm^2^ of slit, in the wavelength range of 400 to 2,500 nm. A single PS layer displays well-resolved Fabry-Pérot fringes in its reflectivity spectrum that is related to the refraction index of the film through the equation *mλ* = 2*nL*, where *m* is the spectral order of the fringe at wavelength λ, *n* is the refractive index of the porous film, and *L* is the thickness of the film [18]. The value *nL* from this equation is regularly referred as OT of the porous layer and is obtained after applying the fast Fourier transform on the inverse of specular reflectance spectrum [21].

Fourier transform infrared (FTIR) spectrometer Varian 660 IR was used to identify the functional groups in thiol-functionalized PS. To determine the presence of As, a Thermo Scientific K-Alpha X-ray photoelectron spectroscopy (XPS) was used.

## Results and discussion

### Scanning electron microscopy (SEM)

**Figure 2:**
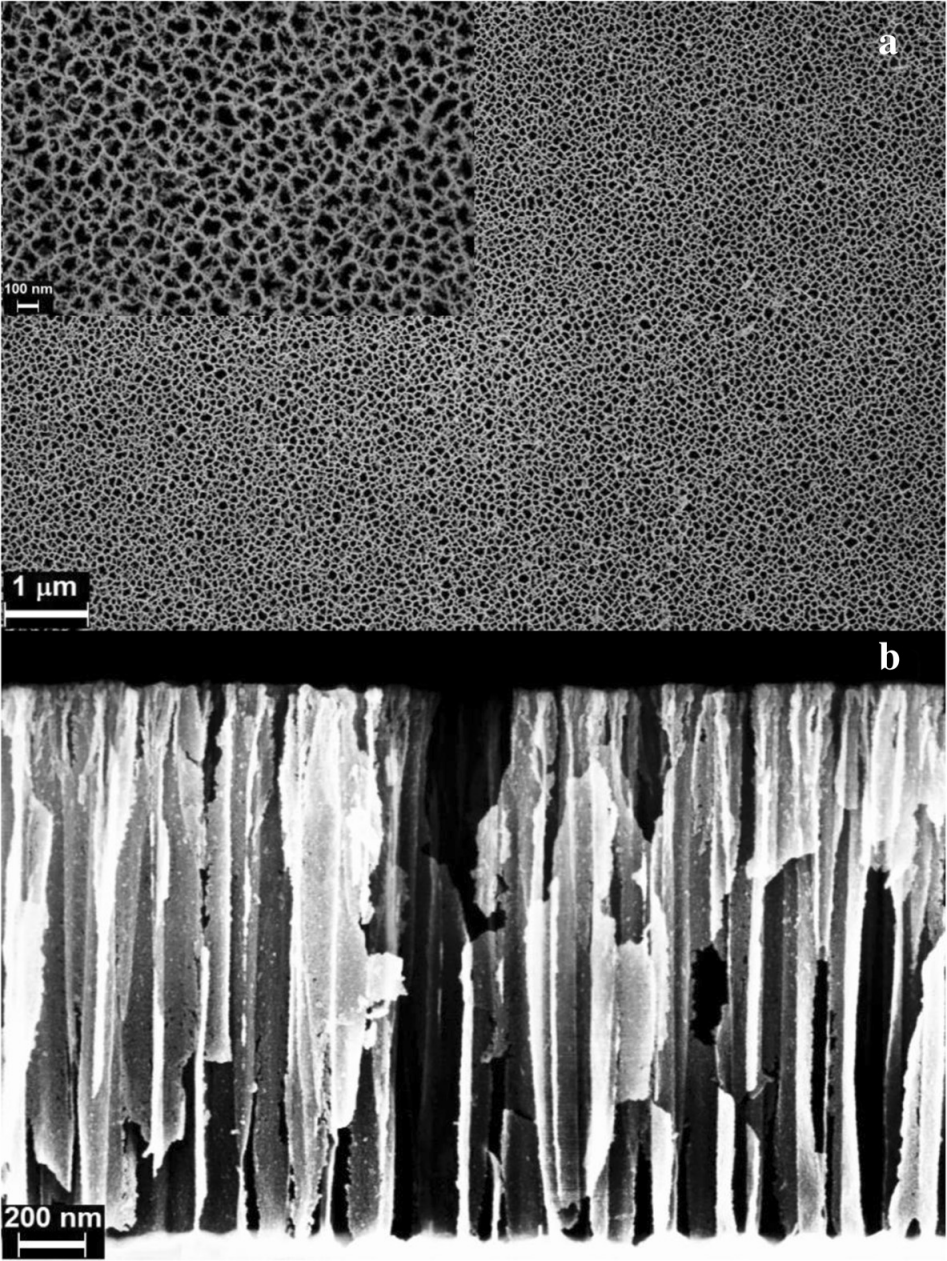
**HRSEM images of the oxidized PS monolayer. (a)** Surface morphology (inset: surface close up) and **(b)** cross sectional view.

Cross sectional and the top view of a typical oxidized PS monolayer is shown in Figure 2. A heterogeneous porous shape typical of electrochemically etched PS is shown in the surface micrograph (Figure 2a) with a porous diameter in a range of 50 to 100 nm (inset picture in Figure 2a). Cross section image in Figure 2b shows a layer thickness of 1.6 μm.

### UV-Vis-NIR spectrophotometry

**Figure 3:**
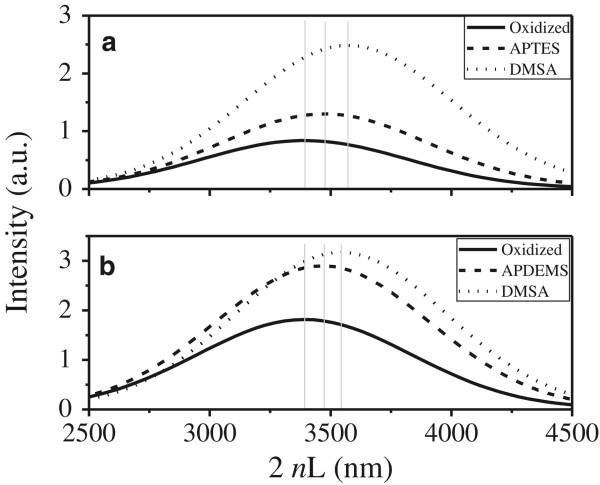
**Fast Fourier transforms of the (specular) reflectance spectrum corresponding to PS monolayers measured after thiol-functionalization.** With **(a)** APTES and **(b)** APDEMS.

The bonding of thiol functionalization on the PS pore walls was monitored by recording the changes in the OT of the porous layer during each modification step, obtained directly from the fast Fourier transform of the (specular) reflectance spectra. Figure 3 shows the OT (2 *nL*) after each modification step. An increase in the refractive index of porous silicon layer due to the chemical attachment of different molecules onto the walls of the pores gives rise to a redshift in the reflectivity spectra, i.e. the refractive index of the film increases as PS walls are covered with different molecules and therefore causes an increase in the OT. In order to compare the optical response of thiol functionalization, two different linkers were used to couple with DMSA: APTES (Figure 3a) and APDEMS (Figure 3b). Table 1 displays the OT shift due to aminosilane and DMSA functionalization. APTES-modified PS surface showed relatively higher values of ΔOT as compared to APDEMS with a relatively lower value measured after the aminosilanization process. The difference between APTES and APDEMS, in the number of ethoxy groups available to form Si-O-Si bond, can be the factor influencing the coverage that each aminosilane reaches and explains the relative change in the ΔOTs. Following a similar trend, it is related to the amount of aminosilane available for the chemical attachment with DMSA and the corresponding ΔOT.

**Table 1:** OT shift obtained after each functionalization

**Aminosilane**	**Redshift (nm)**	
	**Oxidized aminosilane**	**Aminosilane-DMSA**
APTES	124.98 ± 26.31	118.88 ± 15.83
APDEMS	69.29 ± 6.97	68.14 ± 34.27

### FTIR spectroscopy

**Figure 4:**
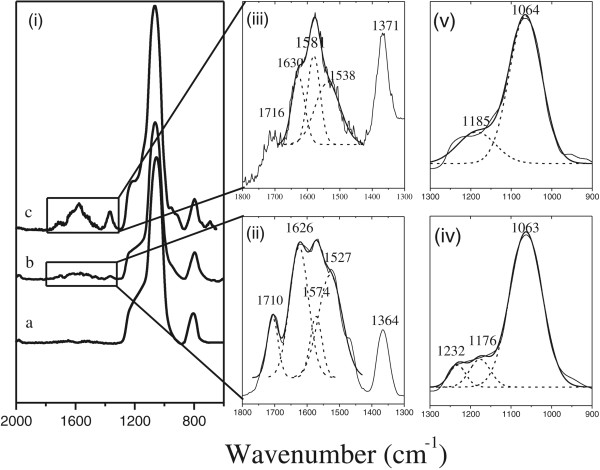
**IR spectra. (i)** PS oxidized shown as **(a)**, PS-DMSA functionalization using APDEMS as linker **(b)**, and PS DMSA-functionalization using APTES as linker **(c)**; **(ii)** deconvoluted peak for amide band, zoom of spectra b; **(iii)** deconvoluted peak of the zoom of spectra c; **(iv)** deconvoluted peak at the 900 to 1,300 cm^−1^ PS-DMSA functionalization using APDEMS as linker; and **(v)** deconvoluted peak at the 900 to 1,300 cm^−1^ PS-DMSA functionalization using APTES as linker.

IR spectroscopy is utilized before and after the functionalization process to monitor the formation of the DMSA-PS device (Figure 4). In Figure 4ia, the most prominent features for the spectra of PS oxidized is located between 1,254 and 967 cm^−1^, where Si-O-Si bonds have been reported [20]. After the thiol functionalization (Figure 4ib,ic using APDEMS and APTES as linkers, respectively), absorption bands were found in the range of 1,800 to 1,300 cm^−1^, an evidence of covalent bond formation. IR spectra reveal a superposition of peaks from 1,800 to 1,400 cm^−1^ and 1,700 to 1,450 cm^−1^, corresponding to APDEMS and APTES respectively. A deconvolution process was necessary to recognize the amide bond absorption bands at 1,630 to 1,626 cm^−1^ and 1,538 to 1,527 cm^−1^ called amide I band (C = O) and amide band II (N-H vibration mode), respectively (Figure 4ii for APDEMS and Figure 4iii for APTES). The third band between the amide bands in the deconvoluted peak at 1,581 to 1,574 cm^−1^ and the bands at 1,371 to 1,364 cm^−1^ correspond to the antisymmetric and symmetric stretching modes of the COO^−^ groups. As evidenced by -COOH band at 1,716 to 1,710 cm^−1^, the attachment of DMSA occurs monofunctionally to some extent. We found the same species in the range of 1,300 to 1,800 cm^−1^ for both functionalization processes, but the concentration of these species in the PS matrix is relatively less for APDEMS than for APTES functionalization, as the absorption band signal is weaker for APDEMS, which confirms the behavior showed by RIFTS analysis. The distinction between the resulting structure after performing the silanization by APTES and APDEMS is attributed to the presence of -CH_3_ group in the APDEMS molecule. Si-CH_3_ band appears in the range of 1,230 to 1,280 cm^−1^. PS-oxidized samples showed high and wide band in the range of 950 to 1,300 cm^−1^, usually assigned to the TO and LO modes of the Si-O-Si asymmetric stretching vibrations. In order to localize the CH_3_ signal in the samples, deconvolution process was necessary. The Figure 4iv shows three deconvoluted peaks at 1,063 cm^−1^ for TO Si-O-Si, 1,176 cm^−1^ for LO Si-O-Si, and 1,232 cm^−1^ that can be assigned to Si-CH_3_ group in the APDEMS functionalized sample. In the APTES functionalized sample, deconvolution of the same peak in the range of 950 to 1,300 cm^−1^ resulted in only two peaks, corresponding to the TO and LO modes of the Si-O-Si group (Figure 4v).

### X-ray photoelectron spectroscopy

**Figure 5:**
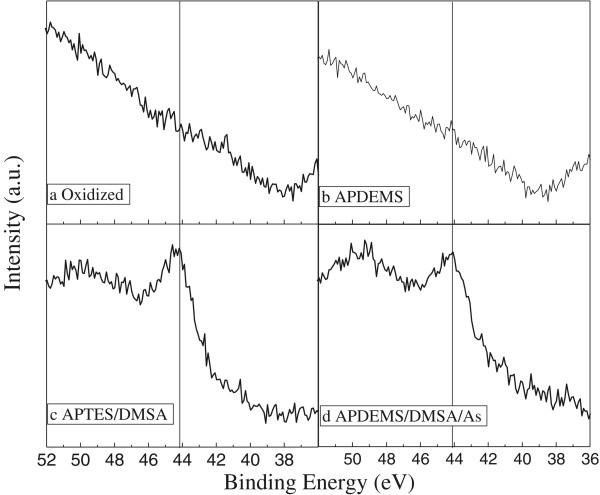
**Arsenic (As) 3d XPS spectra of As (III).** Absorbed on **(a)** oxidized PS sample, **(b)** APDEMS-functionalized PS sample, **(c)** APTES-thiol-functionalized PS sample, and **(d)** APDEMS-thiol-functionalized PS sample.

PS functionalized samples were analyzed with XPS after a wet impregnation with 1 g/l solution of NaAsO_2_. As 3d spectrums of the arsenite adsorbed on porous silicon samples are shown in Figure 5. Oxidized samples and APDEMS-functionalized (Figure 5a,b respectively) and thiol-functionalized APTES linker (Figure 5c) and APDEMS linker (Figure 5d) were tested for a possible As (III) adsorption. For samples without thiol-functionalization, no binding energy peak was found. In contrast, the samples with thiol-functionalization display a clear peak at 44.1 eV for APTES (Figure 5c) and APDEMS (Figure 5d) procedures, which can be assigned to As 3d [22,23]. The As (III) adsorption on PS-DMSA functionalized samples can be understood as follows: the diluted NaAsO_2_ at pH = 2 results in the formation of arsenous acid (H_3_AsO_3_) or As (OH)_3_ which reacts with available thiol functionalities.

## Conclusions

The absence of non-specific As (III) binding on unfunctionalized PS monolayer suggests a preferential adsorption of arsenic on thiol-functionalized PS monolayers. Thiol functionalization using APTES as linker showed a better signal in the functionalization process (specular reflectance and FTIR graphs) as well as in the XPS analysis (a sharper and more intense peak); however, both proved to be efficient linkers for the DMSA immobilization and hence for the As (III) absorption. Such PS adsorption platforms have potential applications as arsenic concentrators and demonstrated many advantages, such as its interferometric-based detection allowed a fast and non-destructive option to appropriately follow the sample functionalization.

## Abbreviations

APDEMS: 3-aminopropyl (diethoxy)-methylsilane; APTES: 3-(aminopropyl) triethoxysilane; DMSA: dimercaptosuccinic acid; EDC: N-(3-dimethylaminopropyl)-N′-ethylcarbodiimide hydrochloride; FTIR: Fourier transform infrared; OT: optical thickness; PS: porous silicon; RIFTS: reflective interferometric Fourier transform spectroscopy; SEM: scanning electron microscopy; XPS: X-ray photoelectron spectroscopy.

## Competing interests

The authors declare that they have no competing interests.

## Authors’ contributions

RFBV carried out all the experimental work. VA conceived the experiments. VA and RFBV analyzed and discussed the results and wrote the final version of the paper. Both authors read and approved the final manuscript.
